# The role of electrical impedance tomography in intensive care units: applications and clinical perspectives

**DOI:** 10.1590/1806-9282.20251019

**Published:** 2026-05-08

**Authors:** Yeliz Bilir, Akın Bilir, Elif Bombacı, Banu Çevik

**Affiliations:** 1 Kartal Dr. Lütfi Kırdar City Hospital, Department of Intensive Care – Istanbul, Turkey.

Dear Editor,

The management of critically ill patients in the intensive care unit (ICU) requires continuous monitoring of physiological parameters to guide therapeutic interventions. Traditional imaging techniques such as chest X-rays and computed tomography provide anatomical information but are limited by their intermittent nature, exposure to radiation, and the need to transport unstable patients^1^.

Electrical impedance tomography (EIT) is an innovative, non-invasive, and radiation-free imaging modality that has brought significant advancements to intensive care practice. Particularly in severe respiratory issues, its ability to provide real-time regional lung monitoring significantly contributes to developing personalized treatment strategies. EIT is based on the measurement of electrical impedance changes across tissues. Electrodes placed around the thorax inject small alternating currents and measure the resulting voltage differences. These data are used to reconstruct cross-sectional images that reflect impedance distribution, which varies with air and fluid content. Since pulmonary impedance changes with ventilation and perfusion, EIT can provide dynamic images of lung function^2^.

In this study, we aim to share the advantages, scientific foundations, and findings from our clinical experiences with EIT under three main headings.

1. Detection of ventilation-perfusion (V/Q) mismatches: One of the most prominent applications of EIT in the ICU is the monitoring of regional ventilation. EIT enables real-time visualization of ventilation and perfusion imbalances, providing insights into adequate gas exchange, perfusion, and shunt status of the lungs^1,2^.

In our clinical practice, we detected a significant V/Q mismatch early in a patient recovering from pneumonia+effusion using EIT. Before observing any evident clinical deterioration, the patient was diagnosed with ventilator-associated pneumonia. We swiftly optimized our treatment strategy and successfully stabilized the patient’s respiratory condition (Figure 1).

**Figure 1.**
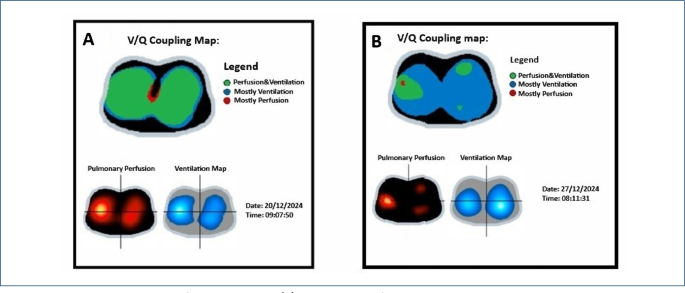
Case with ventilation-perfusion mismatch. **(A)** Ventilation-perfusion coupling image demonstrating regional mismatch. **(B)** Ventilation and perfusion distribution maps obtained by Electrical Impedance Tomography.

2. PEEP titration and lung recruitment: EIT is an effective tool for personalizing positive end-expiratory pressure (PEEP) levels and detecting lung collapse or hyperdistension. In particular, it facilitates optimal PEEP adjustment in acute respiratory distress syndrome (ARDS) patients without causing ventilator-induced lung injury^3,4^.

In one of our ARDS patients, we achieved better lung aeration and oxygenation through personalized ventilation management and the recommended optimal PEEP (Figure 2). This experience reaffirmed the critical role of EIT in personalized treatment management.

**Figure 2:**
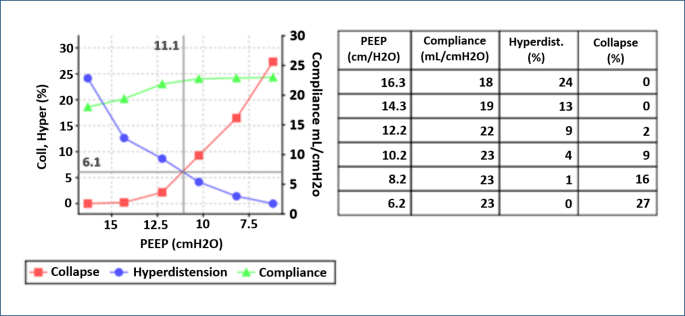
Positive end-expiratory pressure titration graph suggested by the device for the patient.

3. Detection of pendelluft and asynchrony: EIT helps identify asynchrony during mechanical ventilation, enabling more effective patient management and optimizing ventilation strategies^5,6^. With appropriate PEEP and tidal volume adjustments, we observed improvements in lung aeration and homogeneity in real time (Figure 3).

**Figure 3:**
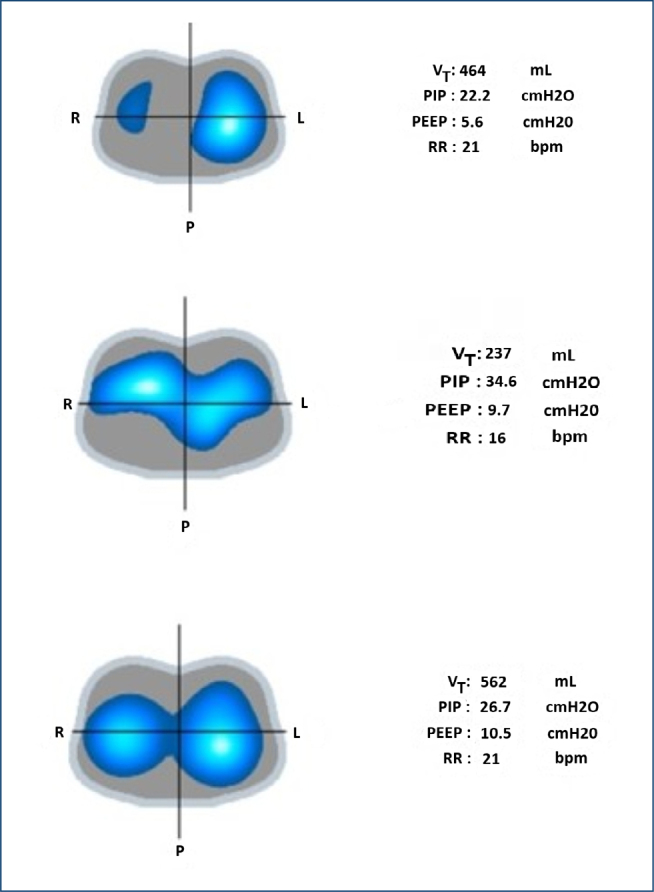
Real-time ventilation strategies in asynchrony cases.

In conclusion, EIT represents a promising tool in intensive care medicine, offering non-invasive, dynamic insights into lung function that are not accessible through traditional imaging. Additionally, EIT is a highly effective tool for improving patient outcomes and enhancing patient safety. We believe that routine use of EIT in intensive care units should be encouraged, and training healthcare professionals on this technology should be widely promoted. Ongoing research is exploring EIT’s role in cardiac monitoring, neurocritical care, and sepsis management. With growing evidence, EIT could become a standard component of personalized, data-driven critical care.

## Data Availability

The datasets generated and/or analyzed during the current study are available from the corresponding author upon reasonable request.
